# HIV Pre-Exposure Prophylaxis Cascade Stages Among Men Who Have Sex With Men With Sexually Transmitted Infections in China: Multicenter Cross-Sectional Survey Study

**DOI:** 10.2196/65713

**Published:** 2024-12-30

**Authors:** Xue Yang, Wenting Kang, Zhuoer Zhang, Houlin Tang, Dapeng Zhang, Lijun Sun, Zaicun Li, An Liu

**Affiliations:** 1 Beijing Huilongguan Hospital, Beijing, China; 2 Chinese Association of STD and AIDS Prevention and Control, Beijing, China; 3 School of Public Health, Capital Medical University, Beijing, China; 4 National Center for AIDS/STD Control and Prevention, Chinese Center for Disease Control and Prevention, Beijing, China; 5 Beijing Youan Hospital, Capital Medical University, Beijing, China

**Keywords:** HIV, men who have sex with men, MSM, pre-exposure prophylaxis, PrEP, sexually transmitted infection, STI

## Abstract

**Background:**

There is limited literature available regarding the knowledge and use of HIV pre-exposure prophylaxis (PrEP) among men who have sex with men (MSM) with sexually transmitted infections (STIs).

**Objective:**

This study’s objective was to explore the HIV PrEP cascade stages (knowledge, willingness to use, and use) among MSM with STIs in China, in order to promote the implementation of PrEP in this population.

**Methods:**

A cross-sectional study was conducted using a respondent-driven sampling method in 19 cities in China, from January to August 2022. The study collected data on demographics, behaviors, and PrEP cascade stages from participants who were not infected with HIV and who self-reported being recently infected with STIs. After using chi-square tests or Fisher exact tests to analyze differences in the knowledge of PrEP, willingness to use PrEP, and PrEP use across different variables, multivariate logistic regression was used to analyze the influences of the different variables on PrEP cascade stages.

**Results:**

By August 2022, following screening and exclusion, a total of 1329 MSM were included in the study. Among them, 85.55% (n=1137) had heard of PrEP, 81.57% (n=1084) expressed their willingness to use PrEP if engaging in high-risk HIV behaviors, 70.58% (n=938) had consulted a health care professional about PrEP, 62.98% (n=837) reported having used PrEP, and 46.35% (n=616) possessed a basic understanding of PrEP. The results of multivariate logistic regression analyses showed that the same factors significantly influenced both knowledge of PrEP and willingness to take PrEP, including age, education, marital status, income, condom usage, participation in group sex, HIV status of the most recent male sexual partner, and postexposure prophylaxis (PEP) usage. The factors significantly related to the PrEP use included income, engagement in commercial sex, participation in group sex, HIV status of the most recent male sexual partner, new drug usage, and PEP usage.

**Conclusions:**

MSM with STIs were engaged with the PrEP cascade stages at a relatively high rate, but they lacked an understanding of PrEP and underestimated HIV risk. The prevalence of having a basic understanding of PrEP was lower than PrEP usage, and this suboptimal awareness could impede PrEP efficacy and lead to risk compensation.

## Introduction

The proportion of HIV transmission through homosexual sex in China has substantially increased, rising from 2.5% in 2006 to 25.6% in 2022 [1]. According to data from the Chinese Center for Disease Control and Prevention, the prevalence of HIV infection among men who have sex with men (MSM) in China is on the rise, increasing from 6.9% in 2019 to 8% in 2020 [2]. Against this backdrop, the HIV epidemic among MSM remains uncontrolled despite the government providing various HIV prevention methods, such as condoms, HIV testing, and antiretroviral therapy (ART) [3]. Therefore, in order to achieve the goal of ending the AIDS epidemic by 2030, the promotion of biomedical preventive measures is indispensable.

Pre-exposure prophylaxis (PrEP) is a novel biomedical form of HIV prevention in which oral antiretrovirals (most commonly a combination of tenofovir and emtricitabine) are taken by individuals with a high risk of HIV acquisition to prevent infection, and it has been shown to be 99% effective [4,5]. PrEP services in China are still under development and the current state of PrEP use is characterized by high awareness, high willingness to use, and low use. Among MSM, 43.1%‐76.5% have heard of PrEP [6,7], 60.1%‐75.7% are willing to use PrEP [6,8], and 3.3%‐18.2% have used PrEP [9,10]. The results of a cross-sectional study conducted in 6 cities across China in 2021 showed that among the 1865 high-risk MSM who had heard of PrEP, the proportions of those who were willing to use PrEP, had knowledge of PrEP, and had previously used PrEP were 96.7%, 24.7%, and 22.4%, respectively [11]. In recent years, the accessibility and affordability of PrEP in China have significantly improved. Although PrEP medications are not yet covered by medical insurance, the introduction of domestic generic drugs in early 2021 has reduced the monthly cost to less than 500 renminbi (US $69). Additionally, MSM can access PrEP services and medications through various channels, including in-person health care facilities, web-based apps, and some pharmacies through the internet.

In China, sexually transmitted infections (STIs) other than HIV also impose a heavy disease burden on MSM [12]. Syphilis is almost twice as prevalent as HIV among MSM in China, and remains an epidemic with a prevalence of 13.5% and incidence of 9.6/100 person-years [13-15]. The result of a meta-analysis showed that the prevalence of STIs among MSM were 6.3% (95% CI 3.5%‐11.0%) for chlamydia, 1.5% (95% CI 0.7%‐2.9%) for genital warts, 1.9% (95% CI 1.3%‐2.7%) for gonorrhoea, 10.6% (95% CI 6.2%‐17.6%) for herpes simplex virus-2 and 4.3% (95% CI 3.2%‐5.8%) for *Ureaplasma urealyticum* [14]. Whether or not one has STIs is also one of the criteria for determining a high-risk of HIV infection in MSM [11]. The knowledge of PrEP, willingness to use PrEP, and PrEP usage among MSM with STIs is unknown.

In addition, PrEP decreases users’ risk of HIV infection but has no effect on the transmission of other STIs [12]. The World Health Organization has recognized the rising burden of STIs and called for the integration of STI treatment and prevention services into HIV PrEP programs [16]. There is a concern that PrEP use could be related to increases in bacterial STIs [17]. A recent systematic review and meta-analysis of studies among PrEP users from high-income and low-and-middle-income countries reporting on the combined outcome of chlamydia, gonorrhea, or syphilis infections reported a prevalence of 23.9% and an incidence of 72.2/100 person-years [18]. According to previous studies, the incidence of syphilis among MSM taking PrEP is 44.6 times higher than MSM who are not taking PrEP [19]. Based on the available literature, it is possible to estimate the prevalence of STIs among PrEP users, but it is not possible to obtain information on the PrEP cascade stages among MSM with STIs.

Originally applied to the control of chronic infectious diseases, the cascade model is now being applied internationally to the control of noncommunicable diseases such as hypertension and diabetes [20]. Implementation analyses of cascade effects (ie, analyses of impediments and facilitators at each stage) can help to effectively implement interventions targeting the influencing factors, so as to reduce patient attrition and improve the control rate of the disease at each stage [21]. In this study, the PrEP cascade was divided into 3 stages, based on a cascade model: knowledge of PrEP, willingness to use PrEP, and PrEP use. The aim of this study was to assess the prevalence of each PrEP cascade stage as well as factors influencing each stage among MSM with STIs, to provide evidence for optimizing PrEP intervention strategies and enhancing the uptake of PrEP services in China.

## Methods

### Study Design and Participants

Cross-sectional surveys were conducted from January to August 2022, in 19 cities in China. We selected 19 provincial capital cities in China based on the following criteria: a large MSM population to ensure a sufficient number of target individuals and a relatively high HIV incidence rate, making them relevant for studying HIV PrEP knowledge and use. Additionally, these cities had an established collaborative relationship with us and had experience in implementing similar projects. This survey was conducted using the respondent-driven sampling method [22]. This method was a peer-referral sampling methodology designed to estimate the characteristics of hard-to-reach groups that cannot be sampled randomly. It is frequently used to identify concealed populations at risk of HIV, including MSM. The inclusion criteria for participants were as follows: (1) male individuals aged 18 or older, (2) had sex with men in the past year, (3) were HIV-negative or had an unclear status, and (4) provided informed consent. The exclusion criteria included transgender and nonbinary individuals, as well as individuals with mental disabilities.

### Procedures

From January to February 2022, the questionnaire was designed based on the literature with input from experts at the Chinese Center for Disease Control and Prevention and community-based organizations (CBOs) for MSM. In March, 10 MSM were invited to complete the questionnaire, and it was revised based on their feedback. The questionnaire included basic demographic characteristics, sexual behavior characteristics, questions regarding the PrEP cascade stages, and others. When the project was formally launched, the questionnaire became available in both electronic and paper formats. Methods and results of the electronic questionnaire were reported in accordance with the Checklist for Reporting Results of Internet e-Surveys guidelines [23]. Each city identified a subproject leader responsible for the investigation of the project in that city. From April to July 2022, the subproject leader selected one CBO for MSM in each of the 19 cities as a study site, and staff were trained on the questionnaire process. One MSM in each of the 19 cities was selected as the seed for the survey, and the seed had to meet the following inclusion criteria: (1) having had sex with other MSM; (2) willingness to recruit other MSM; (3) extensive contacts in the MSM community, with preference given to leaders of CBOs; and (4) an HIV-negative status.

Seeds in this project were identified as the heads of CBOs after coordination with the subproject leaders. After completing the written informed consent and questionnaire, each seed received 3 electronic recruitment cards to distribute to 3 close MSM contacts, inviting them to participate in the survey. Participants who completed the survey also received 3 recruitment cards. The task was considered complete when each electronic recruitment card successfully recruited 3 MSM. The questionnaires had to be completed at the project site (CBO advisory office) under the supervision of the subproject leader. Once the questionnaires were completed, the subproject leader at each site encrypted the electronic versions and sealed the paper questionnaires for mailing to the project leader. Project leaders examined the database, verified the completeness and coherence of the questionnaires, and contacted the subproject leader of each site to identify and address any gaps in the questionnaires. The sample size was estimated by the cross-sectional survey formula N=(*Z*^2^_1-a/2_P[1-P])/d^2^. Based on the rate of PrEP use among high-risk MSM of 22.4% [11], the required sample size for this study was 1324.

### Measures

The participant’s history of STIs was determined based on the question, “Have you been diagnosed with STIs in the past 6 months? (For example, chlamydia, gonorrhea, syphilis, in addition to HIV, etc).” In order to obtain information on MSM who were recently infected with STIs, a time limit of 6 months was set. Selecting yes for this question suggested a recent STI. Knowledge of PrEP was assessed by the question, “Have you ever heard of HIV PrEP?” Willingness to use PrEP was measured with the question, “Would you use PrEP if you were about to engage in high-risk HIV behavior?” PrEP counseling was evaluated based on the question, “Have you actively consulted with health care professionals or others about HIV PrEP?” PrEP use was determined by the question, “Have you ever used HIV PrEP?” Selecting yes for each of the above questions indicated engagement with the corresponding PrEP cascade stage.

Questions for determining whether a person had a basic understanding of PrEP included the following: (1) What do you think is the function of HIV PrEP? (2) Who do you think needs HIV PrEP? (3) Do you know how to take HIV PrEP? The correct answers to the 3 questions were HIV prevention (1 point); MSM, sex workers, and drug users at higher risk of HIV infection (1 point); and take daily (1 point) or take a double dose 2 hours before sex and then one pill 24 and 48 hours later (2-1-1 regimen; 1 point). Each correct response was given a score of 1, with a maximum total score of 4. Obtaining a perfect score was considered as having an understanding of PrEP.

### Ethical Considerations

All participants in this study were required to mark “agree” on the paper informed consent form or click the “yes” button on the electronic informed consent form; otherwise, the questionnaire would be considered invalid. The responses were collected anonymously to ensure privacy. This study was approved by the Institutional Review Board of the National Center for AIDS/STD Control and Prevention, Chinese Center for Disease Control and Prevention (Approval No. X220314678). Upon questionnaire completion, participants received 50 renminbi (RMB), or US $7, and were reimbursed for taxi fares if they traveled for more than 30 minutes. Participants were also provided with free counseling services.

### Data Analysis

Frequency distributions were used to illustrate the sociodemographic features. Bar charts were used to observe the proportional changes in PrEP cascade stages, which also included PrEP counseling and understanding PrEP. Next, proportional differences in the PrEP cascade stages were analyzed using *χ^2^* tests or Fisher exact tests across different behavioral variables. Finally, the influences on PrEP cascade stages among MSM with STIs were analyzed using multivariate logistic regressions. A McFadden pseudo*-R*² was used to evaluate the goodness-of-fit of the model. An *R*² value close to 0.2 or higher was generally considered indicative of a reasonable fit. Statistical analysis was performed using R 4.3.2 (R Core Team). The complete statistical test was a 2-sided test with a significance level of *α*=.05.

## Results

### Sociodemographic Characteristics

**Figure 1.**
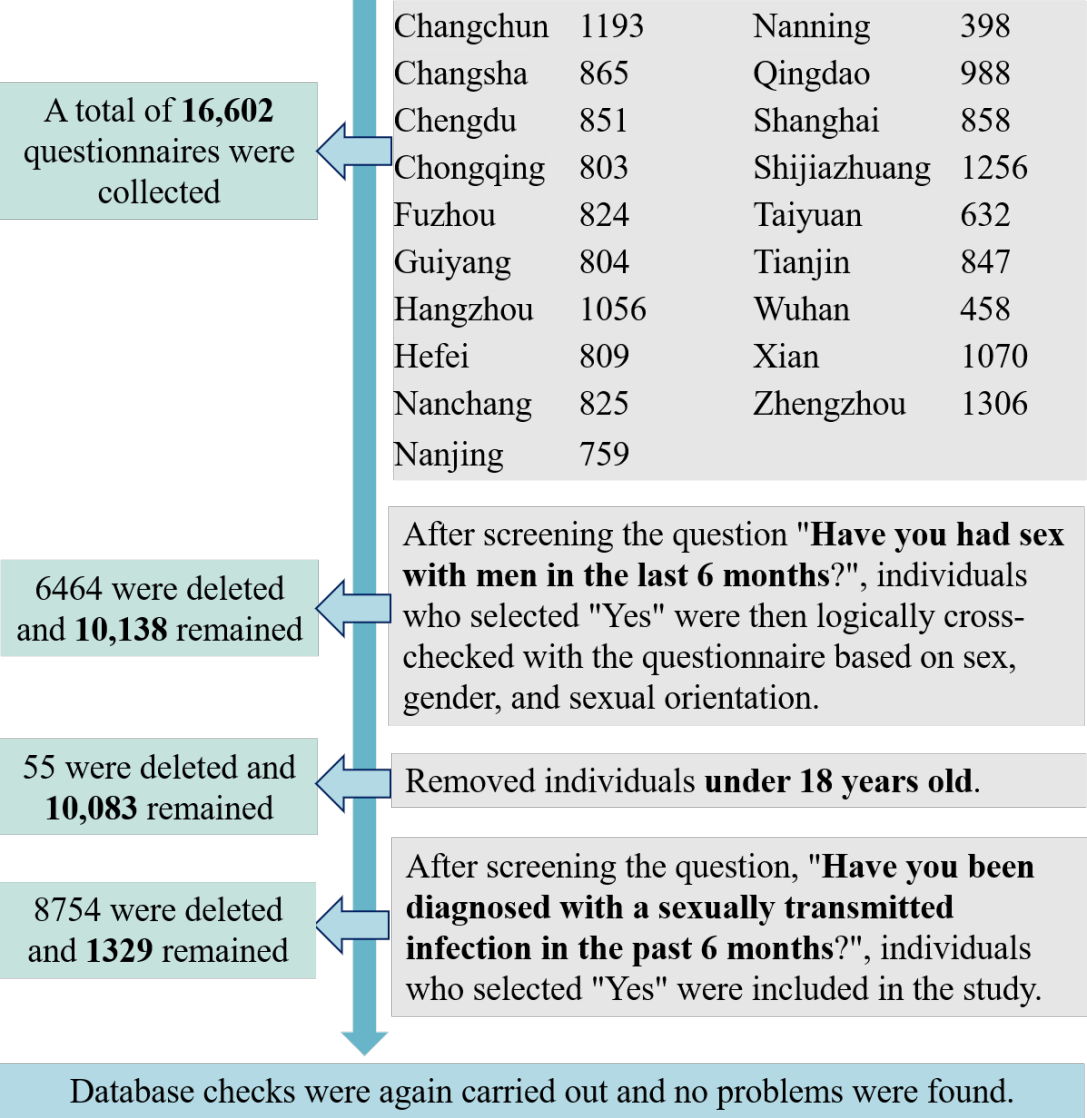
Data for the questionnaire review process.

A total of 16,602 questionnaires were collected from 19 cities, and after being reviewed, 1329 questionnaires were included in the final analysis, which was carried out in accordance with Figure 1. The mean age of the 1329 participants was 29.58 (SD 7.81) years, and 94.88% (n=1261) of participants were of Han ethnicity. Roughly 72.91% (n=969) were local residents according to household registration, 59.29% (n=788) had a diploma or degree, 65.09% (n=865) were unmarried, and 62.3% (n=828) had a full-time job. Furthermore, 12.49% (n=166) of participants identified as bisexual. About half of the participants (n=667) had an average monthly income of less than 5000 RMB (US $687) and one in ten participants had an average monthly income of more than 10,000 RMB (US $1373). Details of the characteristics of MSM with STIs are shown in Table 1.

**Table 1.** Characteristics of MSM^a^ with STIs^b^ in 19 cities in China.

Variables	Total participants (n=1329), n (%)	
Age (years)		
<24	301 (22.65)	
24-28	319 (24)	
29-31	319 (24)	
≥32	390 (29.35)	
Ethnicity		
Han ethnicity	1261 (94.88)	
Other ethnicities	68 (5.12)	
Domicile		
This city^c^	969 (72.91)	
Out of province/city	360 (27.09)	
Education		
Junior high or below	149 (11.21)	
High or technical school	262 (19.71)	
Diploma or degree	788 (59.29)	
Master degree or above	130 (9.78)	
Marital status		
Unmarried	865 (65.09)	
Married	259 (19.49)	
Cohabiting (living together)	170 (12.79)	
Divorced/widowed	35 (2.63)	
Job		
Full-time job	828 (62.3)	
Part-time job	174 (13.09)	
Student	131 (9.86)	
Unemployed	128 (9.63)	
Retired/other	68 (5.12)	
Average monthly income^d^		
<5000 RMB^e^	667 (50.2)	
≥5000-10,000 RMB	529 (39.8)	
≥10,000 RMB	133 (10)	
Bisexual		
Yes	166 (12.49)	
No	1163 (87.51)	

a MSM: men who have sex with men.

b STI: sexually transmitted infection.

c City where the questionnaire was administered.

d A currency exchange rate of 1RMB=US $0.1373 is applicable.

e RMB: renminbi.

### PrEP Cascade Stages

The proportions of participants that had knowledge of PrEP, a willingness to use PrEP, and used PrEP were 85.55% (1137/1329), 81.57% (1084/1329), and 62.98% (837/1329), respectively. A total of 70.58% (938/1329) of the participants had consulted health care professionals regarding HIV PrEP. Of the participants who had heard of PrEP, 69.31% (788/1137) were aware of the function of PrEP and 72.91% (829/1137) knew the eligible population for PrEP. The proportions of participants who knew that PrEP can be taken daily or taken on-demand were 74.32% (845/1137) and 65.96% (750/1137), respectively. Details regarding the participants’ understanding of PrEP are provided in Multimedia Appendix 1. According to our definition, a total of 46.35% (616/1329) of participants demonstrated a basic understanding of PrEP.

**Figure 2.**
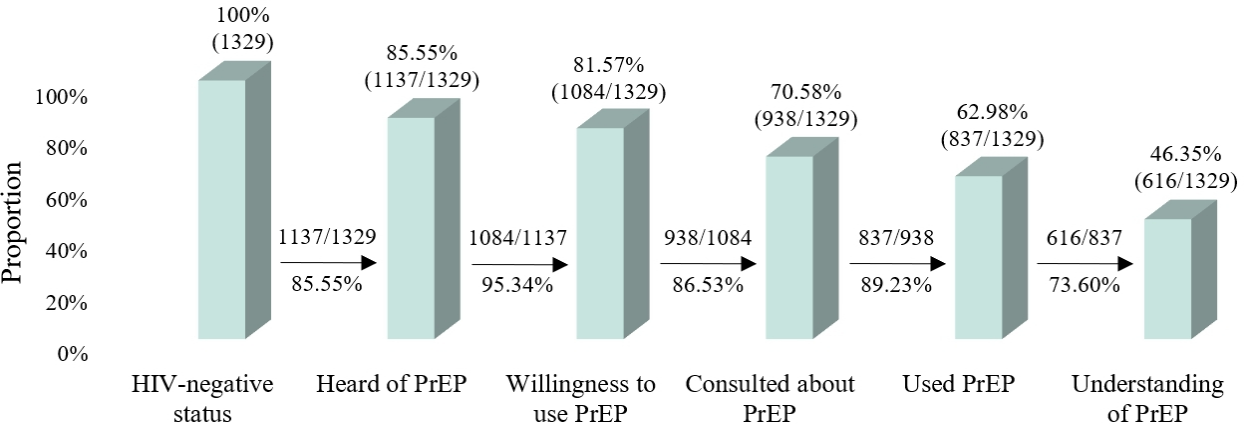
PrEP cascade analyses for HIV-negative men who have sex with men with sexually transmitted infections in 19 cities in China. PrEP: pre-exposure prophylaxis.

As shown in Figure 2, the data above the bars indicate the proportion of HIV-negative MSM by the different variables, while the data between the bars indicate the proportion of the PrEP cascade stage in relation to the previous stage. There was a decreasing progression in the knowledge of PrEP, willingness to use PrEP, consultation about PrEP, usage of PrEP, and understanding of PrEP. The proportion of HIV-negative participants who had heard of PrEP, those who had heard of PrEP and were willing to use it, those who were willing to use PrEP and had been counseled about it, and those who had been counseled about PrEP and had used it were all over 85%. The proportion of participants who had used PrEP and had an understanding of PrEP was 73.6% (616/837). Additionally, the proportions of the PrEP knowledge, willingness to use PrEP, and PrEP use cascade stages among MSM with STIs were 85.55% (1137/1329), 95.34% (1084/1137), and 77.21% (837/1084), respectively.

### Behavioral Characteristics and Differences in PrEP Cascade Stages

Among the 1329 participants, approximately one-third (n=452, 34.01%) had a regular partner. Roughly 18.51% (n=246) never used condoms while having sex with men in the past 6 months. Among all participants, 37.32% (n=496) engaged in commercial sex with men in the last 6 months. Additionally, 12.64% (n=168) participated in group sex with men frequently in the past 6 months. Furthermore, 4.29% (n=57) had sex with HIV-positive individuals not undergoing ART treatment in the past 6 months. A majority of the participants (n=912, 68.62%) reported using new drugs such as inhalable amyl nitrite (rush), methamphetamine, cocaine, ketamine, MDMA, or others in the last 6 months. Notably, 93.83% (n=1247) of the participants underwent HIV testing in the last year. Moreover, 64.94% (n=863) of them used postexposure prophylaxis (PEP).

The use of PrEP showed statistical differences across all behavioral variables (all P<.05; see Multimedia Appendix 2 for detailed results). However, PrEP knowledge alone did not result in statistical differences in the number of sexual partners or condom usage (P>.05). Similarly, willingness to use PrEP alone did not result in statistical differences in condom usage (P=.10). Participants in the groups that had heard of, been willing to use, and used PrEP demonstrated higher proportions of multiple high-risk behaviors than those in the groups that had not heard of, were unwilling to use, and had not used PrEP, respectively. These behaviors included having 2 or more sexual partners, engaging in commercial sex with men, participating in group sex frequently, having sex with HIV-positive men who were not on ART, using new drugs while having sex with men, undergoing HIV testing in the past year, and PEP usage. In addition, it is worth noting that among the participants who had heard of PrEP, had a willingness to use PrEP, and had used PrEP, only 54.2%, 55.6%, and 44.9% had an understanding of PrEP, respectively.

### Factors Influencing the PrEP Cascade Stages

The results of the multivariate logistic regressions indicated that the *R*² values for PrEP knowledge, willingness to use PrEP, and PrEP use were 0.240, 0.249, and 0.416, respectively. The results indicated that the factors significantly associated with PrEP knowledge and willingness to use PrEP were the same, including being 29‐32 years old, having a diploma or degree, marital status, earning over 5000 RMB (US $687), using condoms every time during sex with men, engaging in group sex with men, being uncertain of the HIV status of a recent male sexual partner, and using PEP. The factors significantly related to PrEP use included earning over 5000 RMB (odds ratio [OR] 1.85, 95% CI 1.29-2.65), having commercial sex with men (OR 2.01, 95% CI 1.27-3.19), engaging in group sex with men occasionally (OR 1.83, 95% CI 1.18-2.85), being unclear of the HIV status of a recent male sexual partner (OR 0.58, 95% CI 0.41-0.84), using new drugs while having sex with men (OR 2.21, 95% CI 1.54-3.16) and using PEP (OR 12.31, 95% CI 8.89-17.21). More details are shown in Table 2.

**Table 2.** Multivariate logistic regression analysis for the PrEP^a^ cascade stages among MSM^b^ with STIs^c^ in 19 cities in China.

Variables	Knowledge of PrEP		Willingness to use PrEP		PrEP use	
	OR^d^ (95% CI)	*P* value	OR (95% CI)	*P* value	OR (95% CI)	*P* value
Age (years) (vs <24 years)						
24‐29	1.09 (0.53‐2.21)	.82	1.00 (0.53‐1.87)	.99	0.94 (0.56‐1.60)	.83
29‐32	0.33 (0.17‐0.62)	<.001	0.29 (0.16‐0.50)	<.001	0.61 (0.36‐1.05)	.07
≥32	0.74 (0.38‐1.40)	.36	0.72 (0.40‐1.29)	.28	0.74 (0.43‐1.25)	.26
Education (vs junior high or below)						
High or technical school	1.16 (0.66‐2.04)	.61	1.42 (0.83‐2.43)	.20	0.68 (0.38‐1.20)	.19
Diploma or degree	1.74 (1.03‐2.90)	.04	2.08 (1.28‐3.40)	.003	0.70 (0.41‐1.17)	.18
Master degree or above	1.58 (0.80‐3.18)	.19	1.53 (0.81‐2.92)	.19	0.85 (0.44‐1.66)	.64
Marital status (vs unmarried)						
Married	0.68 (0.43‐1.10)	.12	0.62 (0.40‐0.96)	.03	1.01 (0.65‐1.58)	.97
Cohabiting (living together)	0.50 (0.30‐0.84)	.008	0.39 (0.24‐0.62)	<.001	0.75 (0.46‐1.24)	.26
Divorced/widowed	0.36 (0.15‐0.88)	.02	0.33 (0.14‐0.77)	.009	1.60 (0.61‐4.19)	.34
Job (vs full-time job)						
Part-time job	0.80 (0.47‐1.38)	.41	0.94 (0.57‐1.58)	.82	1.55 (0.92‐2.65)	.11
Student	1.43 (0.65‐3.46)	.40	1.19 (0.60‐2.47)	.63	0.79 (0.44‐1.43)	.44
Unemployed	0.84 (0.44‐1.71)	.62	1.04 (0.56‐2.00)	.91	1.18 (0.66‐2.13)	.58
Retired/other	0.71 (0.36‐1.48)	.35	0.79 (0.41‐1.60)	.50	1.62 (0.81‐3.22)	.17
Average monthly income^e^ (vs <5000 RMB^f^)						
≥5000 RMB	1.85 (1.22‐2.82)	.004	1.70 (1.16‐2.50)	.006	1.85 (1.29‐2.65)	<.001
Bisexual (vs no)						
Yes	1.44 (0.80‐2.73)	.24	1.55 (0.90‐2.77)	.13	0.84 (0.52‐1.39)	.50
Number of men they have had sex with^g^ (vs 1)						
≥2	1.02 (0.70‐1.47)	.94	1.19 (0.84‐1.68)	.33	0.88 (0.62‐1.23)	.45
Use of condoms during sex with men^g^ (vs never)						
Sometimes	1.34 (0.82‐2.18)	.24	1.40 (0.89‐2.18)	.15	0.68 (0.43‐1.06)	.09
Every time	1.70 (1.02‐2.84)	.04	1.95 (1.22‐3.14)	.006	0.91 (0.57‐1.45)	.69
Had commercial sex with men^g^ (vs no)						
Yes	0.86 (0.50‐1.51)	.59	0.70 (0.42‐1.15)	.16	2.01 (1.27‐3.19)	.003
Frequency of group sex with men^g^ (vs never)						
Occasionally	3.08 (1.77‐5.59)	<.001	3.04 (1.84‐5.16)	<.001	1.83 (1.18‐2.85)	.007
Frequently	4.02 (1.55‐12.22)	.008	3.10 (1.40‐7.45)	.008	1.51 (0.75‐3.17)	.26
HIV status of recent male sexual partner^g^ (vs HIV-negative)						
Unclear	0.58 (0.38‐0.87)	.008	0.70 (0.46‐0.98)	.04	0.58 (0.41‐0.84)	.004
HIV-positive sexual partner receiving antiviral therapy	1.41 (0.76‐2.73)	.28	1.34 (0.78‐2.36)	.30	1.34 (0.81‐2.25)	.26
HIV-positive sexual partner not receiving antiviral therapy	0.45 (0.18‐1.28)	.11	0.80 (0.33‐2.21)	.65	1.48 (0.58‐4.06)	.43
Used new drugs during sex with men^g^ (vs no)						
Yes	0.85 (0.57‐1.25)	.40	0.86 (0.59‐1.25)	.42	2.21 (1.54‐3.16)	<.001
Had HIV testing in the last 1 year (vs no)						
Yes	1.34 (0.70‐2.49)	.37	1.64 (0.90‐2.93)	.10	1.91 (1.01‐3.70)	.050
PEP^h^ use (vs no)						
Yes	3.48 (2.36‐5.20)	<.001	3.41 (2.37‐4.94)	<.001	12.31 (8.89‐17.21)	<.001

a PrEP: pre-exposure prophylaxis.

b MSM: men who have sex with men.

c STI: sexually transmitted infection.

d OR: odds ratio.

e A currency exchange rate of 1 RMB=US $0.1373 is applicable.

f RMB: renminbi.

g Indicated for the relevant sexual behavior that had occurred in the past 6 months.

h PEP: postexposure prophylaxis.

## Discussion

### Principal Findings

This study found that MSM with STIs exhibit a high knowledge of PrEP and willingness to use PrEP but exhibit a low usage of PrEP. However, the rate of MSM with STIs that had an understanding of PrEP was unexpectedly lower than the rate of PrEP usage. The results showed that the proportion of participants that had knowledge of PrEP (1137/1329, 85.55%), a willingness to use PrEP (1084/1329, 81.57%), and used PrEP (616/1329, 46.35%) were relatively high among Chinese MSM with STIs. Our findings were compared to a cross-sectional study conducted in 6 cities in China in 2021, in which the proportions of PrEP knowledge (1865/2188, 85.24%) and PrEP use (417/2188, 19.06%) among high-risk MSM in 2021 were lower than this study. However, the proportion of MSM willing to use PrEP (1804/2188, 82.45%) was higher than this study [11]. The willingness to use PrEP in this study was higher than the results of a cross-sectional study in India, where 79.2% of high-risk MSM were willing to use PrEP [24]. The rate of PrEP use was also higher than another study based on monitoring data from MSM consultations in all STI clinics in the Netherlands from 2016‐2021, with 36% of MSM using PrEP in the past 3 months [25]. However, the proportion of participants with a knowledge of PrEP in this study was lower than that among MSM recruited through snowball sampling in Italy, where 87.2% of MSM knew what PrEP was [26]. Although there are no domestic or international studies on the PrEP cascade stages among MSM with STIs, based on the comparisons above with high-risk MSM, MSM with STIs displayed a high willingness to use PrEP but low usage of PrEP, and this is also true for the general MSM population [10].

MSM who use PrEP usually have a deeper understanding of PrEP [11,27]. A noteworthy finding is that the opposite was observed in this study. Considering that only 44.9% of MSM with STIs who had used PrEP had a basic understanding of PrEP, combined with the fact that MSM with STIs who used PrEP had more high-risk sexual behaviors, it is likely that a subset of participants may experience risk compensation while using PrEP. Research exploring the relationship between PrEP use and STIs often involves the concept of risk compensation, wherein PrEP users might engage in more unsafe behaviors, leading to an increase in condomless anal intercourse and consequently an increase in STIs [5]. Literature regarding risk compensation has been mixed, with some studies reporting increased numbers of sexual partners and condomless sex but others finding no meaningful differences among patients after beginning PrEP [28]. Whether the use of PrEP leads to increased STIs in MSM is not clearly established. The results of this study show that MSM with STIs that had knowledge of PrEP, a willingness to use PrEP, or used PrEP exhibited significantly higher sexual risk behaviors compared to MSM with STIs who did not, consistent with the study by Cempaka R et al [29]. This study, as a cross-sectional study, cannot determine a causal relationship, necessitating a future cohort study to address causality.

One of the reasons for low PrEP usage is the misperception of HIV risk among MSM [30]. The level of self-perceived risk of HIV infection had a direct positive effect on PrEP use [10], meaning that individuals who were genuinely at risk for HIV infection might not subjectively perceive themselves as being at risk. This was due to the discrepancy between subjective self-assessment and objective reality [31]. In a recent study, PrEP initiation was associated with an increase in the proportion of MSM reporting never using condoms during anal sex [32]. In this study, 14.23% of MSM with STIs who had not used PrEP reported never using condoms during sex with men in the past 6 months, suggesting that some MSM with STIs might have a misperception of their own risk for HIV infection. In fact, these individuals should consider taking PrEP for HIV prevention. This incorrect self-perception has hindered the promotion of PrEP on a population level, and ultimately, only those who subjectively accepted their risk used PrEP [33]. Considering the current mode of HIV transmission, relying solely on the subjective needs of at-risk populations to decide whether to use PrEP is inadequate.

In addition, it is worth noting that among the participants of this study who had heard of PrEP, had a willingness to use PrEP, and had used PrEP, only 54.2%, 55.6%, and 44.9% had an understanding of PrEP, respectively. This suggests that a basic understanding of PrEP was lacking among MSM with STIs who were engaged with the PrEP cascade stages at that time. Greater participation in events organized by CBOs for MSM can provide more opportunities to access PrEP services [34]. In the future, the role of these CBOs could be strengthened by expanding the scope of PrEP outreach efforts to include guidance on assessing behaviors from an objective point of view (ie, determining which behaviors warrant PrEP use) in addition to providing basic information about PrEP, rather than making subjective decisions about whether or not to use it. This approach will help the MSM with STIs who would benefit from PrEP to begin using it as early as possible.

This study discovered that a higher average monthly income correlated with an increased number of MSM with STIs engaging with each stage of the PrEP cascade. This outcome aligns with the findings of Brooks et al [35]. Higher-income MSM with STIs can afford to purchase PrEP, as it requires payment. Additionally, the results of the multivariate regression analysis revealed that PEP usage was a significant contributing factor influencing engagement with each PrEP cascade stage. Additionally, 88.65% of MSM with STIs who used PrEP in this study also used PEP. Previous studies have recommended that MSM with STIs consistently at risk of HIV exposure consider transitioning from PEP to PrEP [36]. Therefore, it is suggested to include PrEP-related information in HIV prevention and education materials, HIV self-test kits, and PEP products to promote PrEP use among high-risk individuals [10]. Additionally, for MSM with STIs who are at a continuous risk of HIV exposure, it is recommended to incorporate PrEP education and guidance during PEP counseling and follow-up visits, as well as to establish individualized referral services to facilitate a smooth transition and provide the necessary resources and support.

### Limitations

This study had some limitations. Due to the numerous research sites in this study, the relationships between seeds and the recruited participants were not meticulously accounted for. Consequently, the specifics of the recruitment chains and whether the sample composition achieved equilibrium could not be determined. Additionally, the details of the types of STIs contracted by MSM were not carefully investigated in this study due to the limited number of questionnaires. Nonetheless, given the substantial number of participants involved in this study, these factors are unlikely to have a significant effect on the results. In addition, participants had a recall bias when completing the questionnaire, but the researchers limited the questions to a time period of the past 6 months to reduce bias. The cross-sectional design limits the ability to make causal inferences. Also, this study mainly focused on individual-level factors rather than city-level influences, so the cluster effect was not considered in the analysis. The sample sizes across the 19 cities were uneven, and city-level factors such as public health resources, educational initiatives, media exposure, and medical services were not gathered. Therefore, directly evaluating PrEP knowledge and use across different cities is not entirely appropriate. However, to provide more information, the analysis of the variation in PrEP cascade stages among MSM with STIs across different cities is included in Multimedia Appendix 3 for reference. In future research, as the promotion of PrEP expands, it is necessary to recruit a larger sample of MSM with STIs in China that can better reflect the population’s behavioral patterns, to investigate the impact of PrEP on STIs in the real world.

### Conclusion

This cross-sectional study revealed significant information regarding the engagement of MSM with STIs with the PrEP cascade stages across 19 cities in China. A relatively high number of MSM with STIs engaged with the PrEP cascade stages. However, most MSM with STIs who knew of PrEP, were willing to use PrEP, or used PrEP, lacked a basic understanding of PrEP. There were still some MSM with STIs who underestimated their risk of HIV infection. Interestingly, the proportion of MSM who had an understanding of PrEP was lower than the proportion who used PrEP. It is suggested that the understanding of PrEP among MSM with STIs is suboptimal, which could affect the efficacy of PrEP and result in the emergence of risk compensation.

## Supplementary material

10.2196/65713 Multimedia Appendix 1 Pre-exposure prophylaxis understanding in men who have sex with men with sexually transmitted infections in 19 cities in China.

10.2196/65713 Multimedia Appendix 2 Differences in pre-exposure prophylaxis cascade stages among behavioral characteristic variables in men who have sex with men with sexually transmitted infections.

10.2196/65713 Multimedia Appendix 3 The variation in pre-exposure prophylaxis cascade stages among men who have sex with men with sexually transmitted infections across different cities.

## Abbreviations

ART: antiretroviral therapy
CBO: community-based organizations
MSM: men who have sex with men
PEP: postexposure prophylaxis
PrEP: pre-exposure prophylaxis
RMB: renminbi
STI: sexually transmitted infections
