# Supplementary material for: HIV Pre-Exposure Prophylaxis Cascade Stages Among Men Who Have Sex With Men With Sexually Transmitted Infections in China: Multicenter Cross-Sectional Survey Study
Source: JMIR Public Health Surveill. 2024 Dec 30;10:e65713. doi: 10.2196/65713 (PMC11702827; doi:10.2196/65713)
Supplement: Multimedia Appendix 1 [file publichealth-v10-e65713-s001.docx]

***Multimedia Appendix 1***

PrEP knowledge awareness of MSM-STIs in nineteen cities in China^a^

| Variables | Total  (N= 1137) | Proportion (%) |
| --- | --- | --- |
| **What do you think is the function of HIV pre-exposure prophylaxis?** |  |  |
| HIV treatment | 236 | 20.76 |
| HIV prevention | 788 | 69.31 |
| STDs and HIV prevention | 96 | 8.44 |
| Do not know | 17 | 1.50 |
| **Who do you think needs HIV pre-exposure prophylaxis?** |  |  |
| Living with HIV | 176 | 15.48 |
| MSM, sex workers and drug users at higher risk of HIV infection | 829 | 72.91 |
| General population | 113 | 9.94 |
| Do not know | 19 | 1.67 |
| **Do you know how to take pre-exposure prophylaxis? (Multiple choice)** |  |  |
| Take daily | 845 | 74.32 |
| 2-1-1 regime | 750 | 65.96 |
| Do not know | 73 | 6.42 |

^a^ indicated that MSM who have not heard of PrEP have been deleted from this table (n=192).
