# Supplementary material for: HIV Pre-Exposure Prophylaxis Cascade Stages Among Men Who Have Sex With Men With Sexually Transmitted Infections in China: Multicenter Cross-Sectional Survey Study
Source: JMIR Public Health Surveill. 2024 Dec 30;10:e65713. doi: 10.2196/65713 (PMC11702827; doi:10.2196/65713)
Supplement: Multimedia Appendix 2 [file publichealth-v10-e65713-s002.docx]

***Multimedia Appendix 2***

Differences in PrEP cascade services among behavioral characteristic variables in MSM-STIs

| Variables | Total  (N=1329) | Heard of PrEP | | | |  | Willingness to use PrEP | | | |  | PrEP use | | | |
| --- | --- | --- | --- | --- | --- | --- | --- | --- | --- | --- | --- | --- | --- | --- | --- |
|  |  | Yes  (n=1137) | No  (n=192) | χ^2^ | *P* |  | Yes  (n=1084) | No  (n=245) | χ^2^ | *P* |  | Yes  (n=837) | No  (n=492) | χ^2^ | *P* |
| **The numbers of men have had sex with^ab^, n(%)** |  |  |  | 3.287 | .070 |  |  |  | 6.885 | .009 |  |  |  | 4.394 | .036 |
| 1 | 452(34.01) | 375(32.98) | 77(40.10) |  |  |  | 351(32.38) | 101(41.22) |  |  |  | 266(31.78) | 186(37.80) |  |  |
| ≥2 | 866(65.16) | 752(66.14) | 114(59.38) |  |  |  | 725(66.88) | 141(57.55) |  |  |  | 562(67.14) | 304(61.79) |  |  |
| **Using of condoms during having sex with men^b^, n(%)** |  |  |  | 1.703 | .427 |  |  |  | 4.603 | .100 |  |  |  | 13.799 | .001 |
| Never use | 246(18.51) | 204(17.94) | 42(21.88) |  |  |  | 189(17.44) | 57(23.27) |  |  |  | 176(21.03) | 70(14.23) |  |  |
| Sometimes use | 576(43.34) | 497(43.71) | 79(41.15) |  |  |  | 474(43.73) | 102(41.63) |  |  |  | 368(43.97) | 208(42.28) |  |  |
| Use every time | 507(38.15) | 436(38.35) | 71(36.98) |  |  |  | 421(38.84) | 86(35.10) |  |  |  | 293(35.01) | 214(43.50) |  |  |
| **Had commercial sex with men^b^, n(%)** |  |  |  | 41.967 | <.001 |  |  |  | 43.198 | <.001 |  |  |  | 199.060 | <.001 |
| No | 833(62.68) | 672(59.10) | 161(83.85) |  |  |  | 634(58.49) | 199(81.22) |  |  |  | 404(48.27) | 429(87.20) |  |  |
| Yes | 496(37.32) | 465(40.90) | 31(16.15) |  |  |  | 450(41.51) | 46(18.78) |  |  |  | 433(51.73) | 63(12.80) |  |  |
| **Had group sex with men^b^, n(%)** |  |  |  | 82.093 | <.001 |  |  |  | 89.750 | <.001 |  |  |  | 216.370 | <.001 |
| Never | 729(54.85) | 566(49.78) | 163(84.90) |  |  |  | 528(48.71) | 201(82.04) |  |  |  | 331(39.55) | 398(80.89) |  |  |
| Occasionally | 432(32.51) | 409(35.97) | 23(11.98) |  |  |  | 399(36.81) | 33(13.47) |  |  |  | 356(42.53) | 76(15.45) |  |  |
| Frequently | 168(12.64) | 162(14.25) | 6(3.13) |  |  |  | 157(14.48) | 11(4.49) |  |  |  | 150(17.92) | 18(3.66) |  |  |
| **HIV infection of a recent male sexual partner^b^, n(%)** |  |  |  | 33.763 | <.001 |  |  |  | 30.628 | <.001 |  |  |  | 11.640 | <.001 |
| HIV negative | 610(45.90) | 525(46.17) | 85(44.27) |  |  |  | 495(45.66) | 115(46.94) |  |  |  | 394(47.07) | 216(43.90) |  |  |
| Unclear | 386(29.04) | 302(26.56) | 84(43.75) |  |  |  | 288(26.57) | 98(40.00) |  |  |  | 170(20.31) | 216(43.90) |  |  |
| HIV positive sexual partner and receiving antiviral therapy | 276(20.77) | 260(22.87) | 16(8.33) |  |  |  | 252(23.25) | 24(9.80) |  |  |  | 226(27.00) | 50(10.16) |  |  |
| HIV positive sexual partner and not receiving antiviral therapy | 57(4.29) | 50(4.40) | 7(3.65) |  |  |  | 49(4.52) | 8(3.27) |  |  |  | 47(5.62) | 10(2.03) |  |  |
| **Used new drugs during having sex with men^b^, n(%)** |  |  |  | 25.883 | <.001 |  |  |  | 26.279 | <.001 |  |  |  | 246.050 | <.001 |
| Yes | 912(68.62) | 811(71.33) | 101(52.60) |  |  |  | 778(71.77) | 134(54.69) |  |  |  | 703(83.99) | 209(42.48) |  |  |
| No | 417(31.38) | 326(28.67) | 91(47.40) |  |  |  | 306(28.23) | 111(45.31) |  |  |  | 134(16.01) | 283(57.52) |  |  |
| **Had HIV testing in the last 1 year, n(%)** |  |  |  | 4.655 | .031 |  |  |  | 7.611 | .006 |  |  |  | 27.333 | <.001 |
| Yes | 1247(93.83) | 1074(94.46) | 173(90.10) |  |  |  | 1027(94.74) | 220(89.80) |  |  |  | 808(96.54) | 439(89.23) |  |  |
| No | 82(6.17) | 63(5.54) | 19(9.90) |  |  |  | 57(5.26) | 25(10.20) |  |  |  | 29(3.46) | 53(10.77) |  |  |
| **PEP use, n(%)** |  |  |  | 117.090 | <.001 |  |  |  | 122.290 | <.001 |  |  |  | 555.590 | <.001 |
| Yes | 863(64.94) | 805(70.80) | 58(30.21) |  |  |  | 779(71.86) | 84(34.29) |  |  |  | 742(88.65) | 121(24.59) |  |  |
| No | 466(35.06) | 332(29.20) | 134(69.79) |  |  |  | 305(28.14) | 161(65.71) |  |  |  | 95(11.35) | 371(75.41) |  |  |
| **PrEP knowledge awareness, n(%)** |  |  |  |  | <.001^c^ |  |  |  | 201.480 | <.001 |  |  |  | 11.329 | <.001 |
| Yes | 616(46.4) | 616(54.2) | 0 |  |  |  | 603(55.6) | 13(5.3) |  |  |  | 418(49.9) | 198(40.2) |  |  |
| No | 713(53.6) | 521(45.8) | 192(100) |  |  |  | 481(44.4) | 232(94.7) |  |  |  | 419(50.1) | 294(59.8) |  |  |

^a^ indicated that there were 11 records with missing values.

**^b^** indicated whether relevant sexual behavior has occurred in the past 6 months.

^c^  indicated that the fisher test was used.
