# Supplementary material for: HIV Pre-Exposure Prophylaxis Cascade Stages Among Men Who Have Sex With Men With Sexually Transmitted Infections in China: Multicenter Cross-Sectional Survey Study
Source: JMIR Public Health Surveill. 2024 Dec 30;10:e65713. doi: 10.2196/65713 (PMC11702827; doi:10.2196/65713)
Supplement: Multimedia Appendix 3 [file publichealth-v10-e65713-s003.docx]

***Multimedia Appendix 3***

**1 Acceptance of PrEP cascade services of** **MSM-STIs in nineteen cities in China**

The acceptance of PrEP cascade services among MSM-STIs varied by city. Table 1 provides details on the surveyed MSM-STIs, along with the numbers for PrEP knowledge, willingness, and use. Notable discrepancies were observed in the number of MSM-STIs across cities; Shijiazhuang reported 521 individuals, while Taiyuan recorded only 13.

Table 1. PrEP cascade services utilization among MSM-STIs in different cities

|  | Chang  chun | Nanning | Chang  sha | Qingdao | Chengdu | Shanghai | Chongqing | Shi  jiazhuang | Fu  zhou | Tai  yuan | Gui  yang | Tian  jin | Hang  zhou | Wu  han | He  fei | Xi  an | Nan  chang | Zheng  zhou | Nan  jing |
| --- | --- | --- | --- | --- | --- | --- | --- | --- | --- | --- | --- | --- | --- | --- | --- | --- | --- | --- | --- |
| Total, N | 70 | 19 | 172 | 76 | 25 | 33 | 32 | 521 | 33 | 13 | 61 | 37 | 30 | 18 | 30 | 83 | 14 | 18 | 44 |
| Heard of PrEP, n | 58 | 16 | 92 | 45 | 22 | 31 | 28 | 517 | 25 | 11 | 58 | 30 | 26 | 17 | 25 | 73 | 13 | 14 | 36 |
| Willingness to use PrEP, n | 53 | 16 | 70 | 39 | 20 | 30 | 28 | 513 | 24 | 11 | 54 | 29 | 26 | 16 | 25 | 70 | 13 | 14 | 33 |
| PrEP use, n | 23 | 10 | 56 | 28 | 15 | 7 | 11 | 501 | 12 | 6 | 41 | 15 | 11 | 8 | 8 | 52 | 3 | 7 | 23 |

Figure 1 presents the utilization proportions of PrEP cascade services among MSM-STIs in different cities. High acceptance was observed in cities like Shijiazhuang, where 99.23% had heard of PrEP, 98.46% expressed willingness to use it, and 96.16% had used it. In contrast, lower acceptance was observed in Changsha, with 53.49% aware of PrEP, 40.70% willing to use it, and 32.56% having used it.


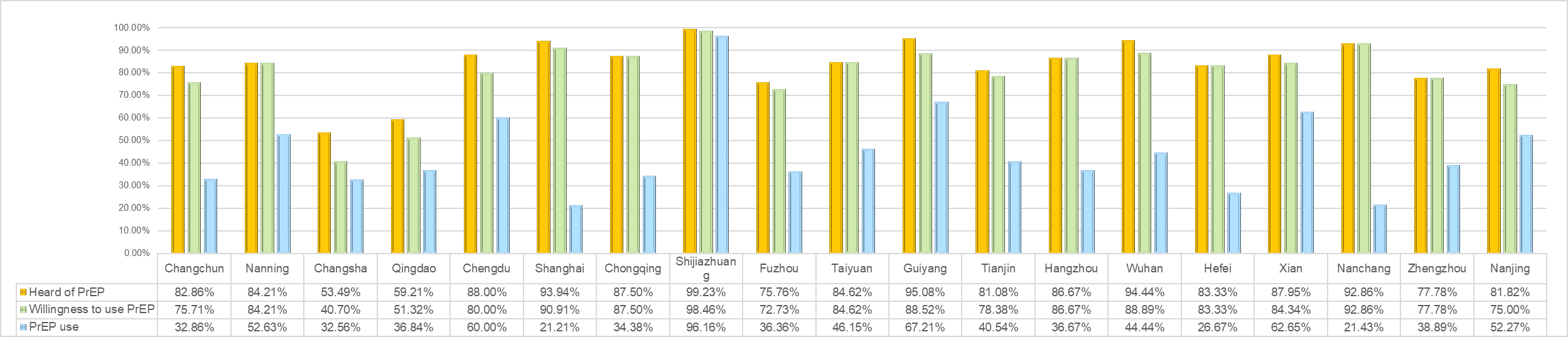
Figure 1. The proportions of PrEP cascade services utilization among MSM-STIs in different cities

**2 The variation in the acceptance of PrEP cascade services among MSM-STIs across different cities**

The purpose of this step was to analyze the differences in the acceptance of PrEP cascade services among MSM-STIs in 19 cities. For the binary variables (knowledge, willingness, and use), we used the “glmer” function of the Generalized Linear Mixed Model (GLMM) to fit the model. Incorporating cities as random effects in the model accounted for the variability in PrEP cascade services uptake across different cities. This stratified analysis helped to identify differences in PrEP cascade services between different cities and controlled for potential confounding factors between cities.

Table 2 detailed the model fitting outcomes. Given the absence of measured city-level factors in this study, such as public health resources, educational initiatives, media exposure, and medical services, a random effects model was deemed appropriate. The variances of the city-specific random intercepts of knowledge, willingness, and use were 0.939, 0.862, 0.933, respectively. The variances significantly above zero indicate substantial variability in PrEP cascade service uptake across cities, suggesting that city-level factors contribute to differences in knowledge, willingness, and use of PrEP.

Table 2. Fitting results of Generalized Linear Mixed Model

| Predictor variables | Hierarchical structure | Goodness of Fit | |  | Random effects | |  | Fixed effects | | |
| --- | --- | --- | --- | --- | --- | --- | --- | --- | --- | --- |
|  |  | AIC | BIC |  | Variance | Std.Dev. |  | Estimate | Z-value | *P*-value |
| Heard of PrEP | Nineteen cities | 873.3 | 883.7 |  | 0.939 | 0.969 |  | 1.902 | 7.491 | <0.001 |
| Willingness to use PrEP | Nineteen cities | 980.7 | 991.0 |  | 0.862 | 0.928 |  | 1.598 | 6.639 | <0.001 |
| PrEP use | Nineteen cities | 1270.7 | 1281.1 |  | 0.933 | 0.966 |  | -0.164 | -0.69 | 0.490 |
